# Oxaliplatin induces pH acidification in dorsal root ganglia neurons

**DOI:** 10.1038/s41598-018-33508-6

**Published:** 2018-10-10

**Authors:** Beatrice Riva, Marianna Dionisi, Alberto Potenzieri, Alessia Chiorazzi, Celia Cordero-Sanchez, Roberta Rigolio, Valentina Alda Carozzi, Dmitry Lim, Guido Cavaletti, Paola Marmiroli, Carla Distasi, Armando A. Genazzani

**Affiliations:** 1 Department of Pharmaceutical Sciences, University of Piemonte Orientale, Via Bovio 6, 28100 Novara, Italy; 2 Experimental Neurology Unit, School of Medicine and Surgery, University of Milano-Bicocca, Via Cadore 48, 20900 Monza, Italy; 3 Young Against Pain Group, Monza, Italy

## Abstract

Oxaliplatin induced peripheral neurotoxicity is characterized by an acute cold-induced syndrome characterized by cramps, paresthesias/dysesthesias in the distal limbs and perioral region, that develops rapidly and lasts up to one week affecting nearly all the patients as well as by long-lasting symptoms. It has been previously shown that pharmacological or genetic ablation of TRPA1 responses reduces oxaliplatin-induced peripheral neurotoxicity in mouse models. In the present report, we show that treatment with concentrations of oxaliplatin similar to those found in plasma of treated patients leads to an acidification of the cytosol of mouse dorsal root ganglia neurons in culture and this in turn is responsible for sensitization of TRPA1 channels, thereby providing a mechanistic explanation to toxicity of oxaliplatin. Reversal of the acidification indeed leads to a significantly reduced activity of TRPA1 channels. Last, acidification occurs also *in vivo* after a single injection of therapeutically-relevant doses of oxaliplatin.

## Introduction

Chemotherapy-induced peripheral neurotoxicity is a dose-limiting toxicity of several anti-cancer agents, including platinum compounds, anti-tubulins and proteasome inhibitors. Management of peripheral neuropathy remains one of the most important unmet clinical needs in oncology practice since it compromises the quality of life and may limit the effectiveness of treatment (if doses need to be adjusted).

Oxaliplatin (OHP) induced peripheral neurotoxicity (OIPN) is characterized by an acute cold-induced syndrome characterized by cramps, paresthesias/dysesthesias in the distal limbs and perioral region, that develops rapidly and lasts up to one week affecting nearly all the patients^1–3^. In addition to these acute symptoms, most patients develop a chronic sensory neurotoxicity with enduring characteristics like ataxia, dysesthesias, paresthesias, burning and lancinating pain, that spreads from toes and fingers with a stocking-and-glove distribution^4^. The chronic symptoms are shared with other platinum-containing chemotherapeutics (*e.g*. cisplatin, carboplatin), while acute toxicity is a peculiarity of OHP. No therapies are available to treat OIPN and patients often suffer from pain and sensory symptoms long after the end of the therapy^5^.

Dorsal root ganglia (DRG) are thought to be the main target of OIPN, although the exact mechanism by which this occurs is still unknown. Since it is well established that intracellular calcium is a crucial second messenger that can induce short and long-term changes in the peripheral nervous system, the hypothesis that intracellular calcium-related events might play an important role in the onset of OIPN has been raised^6,7^, but the mechanism by which changes in calcium signalling would occur are so far unknown. Hyper-excitability of neurons through triggering of voltage-operated sodium channels, which is not in contradiction with the Ca^2+^-hypothesis, has also been postulated as a triggering event^8,9^.

Transient receptor potential channels are non-selective cation channels that detect a vast array of signals. Given that they play an important role in DRG neurons^10^, it is not surprising that some of its family members may be involved in OIPN^11^. For example, OHP-treated TRPA1^−/−^ animals display reduced cold hypersensitivity^12^, an effect which is also observable in TRPM8^−/−^ animals^13^.

In the present study, we set to investigate the relationship between OHP and TRPA1 activation in DRG neurons. We now report that OHP, in DRG neurons, induces cytosolic acidification and TRPA1 sensitization. This sensitization could be reversed if pH was restored to physiological pH, suggesting that it is acidification which leads to TRPA1 sensitization. Furthermore, acidification of intracellular pH was also observed in DRG cells excised from acutely or chronically OHP-treated animals, when measured within 24 hours after injection. In conclusion, our data demonstrate that OHP-induced cytosolic acidification is a key step in TRPA1 sensitization and may be related to acute toxicity.

## Materials and Methods

### Animals and husbandry

BALB/c male mice aged 5–10 weeks upon arrival were employed (Envigo, San Pietro al Natisone, Italy) both for DRG culture preparation and *in vivo* studies. Animals were maintained as previously reported^14^. Care and husbandry of animals were in conformity with the institutional guidelines in compliance with national and international laws and policies. The study plan was approved by the animal Ethics Committee of the University of Milano Bicocca and of the Università del Piemonte Orientale. The procedures were approved by the local animal-health and ethical committee (Università del Piemonte Orientale) and were authorized by the national authority (Istituto Superiore di Sanità; authorization number N. 22/2013). All mice were euthanized under deep isoflurane-induced anaesthesia for cell cultures and with CO_2_ for *in vivo* experiments.

### Chemicals

For *in vivo* studies OHP solution was prepared as reported by Renn and collaborators^15^ and used as previously described^14^.

For *in vitro* studies, OHP (5 mg/mL stock solution, Sigma-Aldrich Inc., Italy), cisplatin (5 mg/mL stock solution, Sigma-Aldrich Inc., Italy), sodium oxalate (10 mg/mL stock solution, Sigma-Aldrich Inc., Italy), capsaicin (1 mM stock solution, Sigma-Aldrich Inc., Italy), icilin (10 mM stock solution, Sigma-Aldrich Inc., Italy), BCTC (10 mM, stock solution, Sigma-Aldrich Inc., Italy), HC-030031 (50 mM stock solution, Sigma-Aldrich Inc., Italy), AITC (allyl isothiocyanate; 50 mM stock solution; Sigma-Aldrich Inc., Italy), BCECF ((2′,7′-Bis-(2-Carboxyethyl)-5-(and-6)-Carboxyfluorescein, Acetoxymethyl Ester, 100 mM stock solution, Life Technologies, Italy), DIDS (4,4′-Diisothiocyano-2,2′-stilbenedisulfonic acid; Sigma-Aldrich Inc., Italy) and nigericin (20 mM stock solution, Life Technologies, Italy) were used. These compounds, with the exception of capsaicin (reconstituted in 100% EtOH), were dissolved in 100% dimethyl sulfoxide (DMSO) and stored at −20 °C, according to manufacturers’ specifications. Working concentrations of these drugs were freshly prepared for each experiment by diluting DMSO or EtOH to 0.1% in milliQ (MilliPore) water.

### Experimental design for the *in vivo* study

For the evaluation of pH in chronically OHP-treated animals, animals (n = 12 in two separate experiments) were injected intravenously in the tail vein with OHP 3.5 mg/Kg (10 mL/Kg) twice a week for 4 weeks, while control mice (n = 12) were left untreated. The neurotoxicity of this chronic schedule and its capacity to mimic Human OIPN have already been reported in detail^15^. In a second experiment that evaluated the acute effect of OHP, eight animals (in two separate experiments) were injected intravenously in the tail vein with a single dose of OHP 3.5 mg/Kg (10 mL/Kg). Animals were sacrificed after 24 hours to investigate acute effects.

### Isolation and Primary Cell Culture of Mouse DRG Neurons

DRG obtained from adult BALB/c mice (5/10-wk-old) were excised and collected in a dish containing cold F12 (Nutrient Mixture F12 Ham) medium (Sigma Aldrich Inc.). Working under a dissecting microscope and using fine forceps, the surrounding membranes were gently teased away from each DRG; nerves and sheath were cut. All de-sheathed DRG were then transferred into a sterile 35 mm dish containing collagenase from Clostridium hystoliticum 0.125% (Sigma Aldrich Inc.) and DNase (Sigma) in F12 (Nutrient Mixture F12 Ham) medium and incubated at 37 °C for 1 h. After incubation, DRG were triturated using a 1000 µl tip. Myelin and nerve debris were eliminated by centrifugation through a bovine serum albumin (BSA) cushion. Cell pellets were re-suspended in Bottenstein and Sato medium (BS) (30% F12 (Nutrient Mixture F12 Ham medium), 40% DMEM (Dulbecco’s Modified Eagle’s medium (Sigma Aldrich Inc., Italy), 30% Neurobasal A medium (Life Technologies, Italy), 100 X N2 supplement (Life Technologies, Italy), penicillin 10 U/mL and streptomycin 100 mg/mL (Sigma Aldrich Inc., Italy), supplemented with Recombinant Human β-NGF, Recombinant Murine GDNF and Recombinant Human NT3 (Peprotech, USA) and plated onto 24 mm glass coverslips pre-coated with laminin (Sigma Aldrich Inc., Italy).

### Real Time Quantitative PCR (QRT-PCR)

Total RNA was isolated from DRG using TRI-Reagent® and reverse transcribed according to the manufacturer’s instructions (Im-Prom-II™ Reverse Transcription System, Promega, WI, USA). cDNA was then stored at −20 °C until further used. qRT-PCRs were performed on 96-well plates (CFX96™ Real-Time PCR Detection Systems, Bio-Rad Inc), in triplicate and florescence intensity assessed using the CFX96™ Real-Time PCR Detection Systems (Bio-Rad Inc.). After an initial denaturation step at 95 °C (10 min), each primer set was used (through 40 cycles of amplification) as follows: mouse TRPV1 5′-CCTGCATTGACACCTGTGAG-3′ forward, 5′-AGAAGATGCGCTTGACAAATC-3′ reverse; mouse TRPM8 5′-TGGAGCCAAAAACTTTGCTT-3′ forward, 5′-TCATCAGGCCGTAGTGAGTG-3′ reverse and mouse TRPA1 5′-TGTCACCCCTTCACATAGCT-3′ forward, 5′-GTGGACATCAAAGCCGTGTT-3′ reverse; 60 °C annealing temperature. Transcripts were normalized to the expression of Ribosomal Protein S18 mRNAs, for each gene, the threshold cycle *(C*_*t*_) was calculated. The *C*_*t*_ of treated cells was compared to the *C*_*t*_ generated by the control cells and *ΔC*_*t*_ was calculated as the difference between *C*_*t*_ values, determined using the equation 2^−ΔCt^.

### Cell lines

Human colorectal carcinoma LS180, HCT116, LoVo, HT29, HCT15 cell lines and SW620 colon cancer cells derived from metastatic site cell lines were obtained from ATCC (Rockville, MD, USA) and were cultured and grown according to the data sheets.

To evaluate viability, the 3-(4,5-Dimethylthiazol-2-Yl)-2,5-Diphenyltetrazolium Bromide (MTT) assay was used. Briefly, cells were plated at concentrations 2.5 × 10^4^ per well in 24 well plates and treated with OHP and Na^+^ oxalate. At the end of the treatments, the MTT reagent was added to cells at a final concentration of 0.25 mg/mL (Sigma-Aldrich Inc., Milan, Italy), for 90 min at 37 °C. Reactions were stopped and the crystals were solubilized by adding isopropyl alcohol/HCL (1:1; vol:vol, Sigma-Aldrich Inc., Milan, Italy), before reading the absorbance at 570 nm, using the multi-plate reader Victor3 V (PerkinElmer, Milan, Italy). pH determinations with BCECF on LoVo and LS180 was performed as described in the relevant section below.

### Fura-2 Ca^2+^ measurements and image analysis

For Ca^2+^ imaging, DRG cultures grown onto 24 mm round cover-slips were loaded with 5 μM Fura-2 AM in presence of 0.02% of Pluronic-127 (both from Life Technologies) and 10 μM sulfinpyrazone (Sigma Adrich Inc., Italy) in Krebs–Ringer buffer (KRB, 135 mM NaCl, 5 mM KCl, 0.4 mM KH_2_PO_4_, 1 mM MgSO_4_, 5.5 mM glucose, 20 mM HEPES, pH 7.4) containing 2 mM CaCl_2_ (30 min, room temperature). Cells were washed and incubated with KRB for other 15 min to allow de-esterification of Fura-2. During the experiments the coverslips were mounted into acquisition chamber and places on the stage of a Leica DMI6000 epifluorescent microscope equipped with S Fluor ×40/1.3 objective. Cells were alternatively excited at 340/380 nm by the monochromator Polychrome IV (Till Photonics, Germany) and the fluorescent signal was collected by a CCD camera (Hamamatsu, Japan) through bandpass 510 nm filter; the experiments were controlled and images analysed with MetaFluor (Molecular Devices, Sunny-vale, CA, USA) software. Data in traces are expressed as 340/380 ratio while to quantify the differences in the amplitudes of Ca^2+^ transients the ratio values were normalized according to the formula ΔF/F0 (referred to as normalized Fura-2 ratio, “Norm. Fura ratio”).

To obtain the size of the neurons, images of fluorescent Fura-2 loaded neurons excited at 380 nm were analysed using ImageJ software (National Institutes of Health, Bethesda, MD, USA, http://imagej.nih.gov/ij). The size was estimated by the average soma diameter, calculated by measuring and averaging the length of the minimum and maximum Feret diameters of the neuronal cell body. TRPA1-positive neurons were determined by their ability to respond to icilin, without any prior selection. The diameter of these neurons is depicted in Suppl. Fig. S2.

### Measurement of intracellular pH in DRG cultures by epifuorescence microscopy with BCECF

DRG cultures or colorectal cancer cells grown onto 24 mm round cover-slips were incubated with 1 μM BCECF (Life Technologies, Italy) in Krebs–Ringer buffer (KRB, 135 mM NaCl, 5 mM KCl, 0.4 mM KH_2_PO_4_, 1 mM MgSO_4_, 5.5 mM glucose, 20 mM HEPES, pH 7.4) containing 2 mM CaCl_2_ (15 min, room temperature). Subsequently, cells were washed and re-suspended in KRB (pH 7.4). A Leica DMI6000 epifluorescent microscope equipped with S Fluor ×40/1.3 objective was used. Cells were alternatively excited at 490/450 nm (monochromator Polychrome IV, Till Photonics, Germany) and the fluorescent signals were collected every 10 seconds (Hamamatsu, Japan); the experiments were controlled and images analysed with MetaFluor (Molecular Devices, Sunny-vale, CA, USA) software. Finally, intracellular pH was calculated by comparing 525/610 nm emission fluorescence ratios with *in vivo* calibration curves obtained by pH equilibration using the proton ionophore nigericin (20 μM) and Intracellular pH Calibration Buffer Kit (pH 7.5-5.5, Life Technologies).

### Measurement of intracellular pH in DRG by flow cytometry with BCECF

After *in vivo* treatment and dissociation, DRG cells were incubated with 1 μM BCECF (Life Technologies, Italy) in Krebs–Ringer buffer (KRB, 135 mM NaCl, 5 mM KCl, 0.4 mM KH_2_PO_4_, 1 mM MgSO_4_, 5.5 mM glucose, 20 mM HEPES, pH 7.4) containing 2 mM CaCl_2_ (15 min, room temperature). Cells were washed, re-suspended in KRB (pH 7.4) and used for flow cytometry (FACSCanto I, Becton Dickinson,). Excitation was performed at 488 nm, using an air-cooled, 20-mW solid-state laser, the emission fluorescence was collected and the FITC and PE median fluorescence values and CV were determined using FACS Diva software. Approximately 10,000 cells were collected in a user-defined gate. A calibration curve was then prepared by suspending cells in high [K^+^] buffers (pH 5.5-6.5-7.5) containing 20 µM of the proton ionophore nigericin (Intracellular pH Calibration Buffer Kit, Life Technologies). Finally, intracellular pH was calculated by comparing FITC/PE nm emission fluorescence ratios with the values obtained by the calibration curve.

### Electrophysiology

Cell attached patch clamp recordings were performed at 22–25 °C on control and OHP-treated neurons with a mean soma diameter < 25 μm. Prior to the gigaseal formation, cells were continuously superfused with a standard physiological solution of the following composition (in mM): NaCl 154; KCl 4; CaCl_2_ 2; MgCl_2_ 1; 4-(2-hydroxyethyl)-1-piperazine ethane sulfonic acid (HEPES) 5; glucose 5.5; NaOH to pH 7.4. The patch electrodes prepared from borosilicate glass capillaries (World Precision Instruments) had a resistance of 5–7 MΩ. In most experiments the pipette solution contained (in mM): CsCl 130, TEACl 20, DIDS 1; 4-(2-hydroxyethyl)-1-piperazine ethane sulfonic acid (HEPES) 10, EGTA 2, BCTC 3 μM, Icilin 1 μM, CsOH to pH 7.4. In a set of experiments, we added 2 mM CaCl_2_ in the absence of EGTA in the patch pipette solution. After seal formation (2–10 GΩ), cells were perfused with a solution containing KCl 150, MgCl_2_ 2, CaCl_2_ 1, EGTA 1.1, HEPES 5, KOH to pH 7.4, in order to set the membrane potential near 0 mV and to prevent intracellular calcium loading. Data were collected and filtered at 1 kHz with an Axopatch 200B amplifier (Molecular Devices, USA) and digitized continuously at a sampling frequency of 1 kHz with PClamp Axoscope software (Molecular Devices, USA). Steady state voltage clamp protocols were applied and digitized at 10 or 20 kHz with PClamp Clampex software Data analysis was performed with OriginPro (OriginLab, USA) and PClamp Clampfit software.

### Statistical analysis

Data are presented as mean ± SEM or Median and IQR. Data points in the single channel current-voltage graphs represent mean ± S.D., estimated by fitting the amplitude histograms to a sum of Gaussian functions (OriginPro 9.1, OriginLab USA). The normality of data distributions was assessed using Shapiro–Wilk test. Parametric (unpaired t-test and One-way analysis of variance (ANOVA) followed by Tukey’s post-hoc) or non parametric (Mann-Whitney U test and One-way Kruskal-Wallis H test followed by Dunn’s post-hoc) statistical analysis was used for comparisons of data. All statistical assessments were two-sided and a value of P < 0.05 was considered statistically significant. Statistical analyses were performed using GraphPad Prism software (GraphPad Software, Inc., USA). In *in vitro* experiments, the n number was calculated on the number of cells, and the number of independent experiments (defined as cultures from separate animals performed on different days) is given in the respective figure legends.

## Results

### Toxicity of OHP in dorsal root ganglia neuronal cultures

Most *in vitro* studies so far performed to elucidate the mechanism of neurotoxicity have used concentrations of OHP in the range of 5–50 µg/mL^16,17^. In the present work, three different concentrations (0.1, 1, 10 µg/mL) of OHP were initially tested. Briefly, DRG neurons were exposed for 6 or 48 hours to OHP. Experiments were carried out after 48 hours of the treatment. Therefore, for the 6 hours exposure, cells were washed and grown for a further 42 hours in normal medium in the absence of OHP before the experiments. Immunostainings with GAP43^17^ showed well-developed neurites when cells were exposed for only 6 hours with the lowest concentration of OHP (0.1 µg/mL), while other treatments showed a marked reduction both in neurite length and in the number of surviving neurons (Suppl. Fig. S1). The concentration of 0.1 µg/mL for 48 hours leads only to minor alterations and was therefore also carried forward for calcium-signalling characterization together with the 6 hour treatment. It should be noted that the reported plasma concentrations of OHP in patients are in the range of 0.1 µg/mL^18^.

### OHP modifies responses to capsaicin and icilin

**Figure 1:**
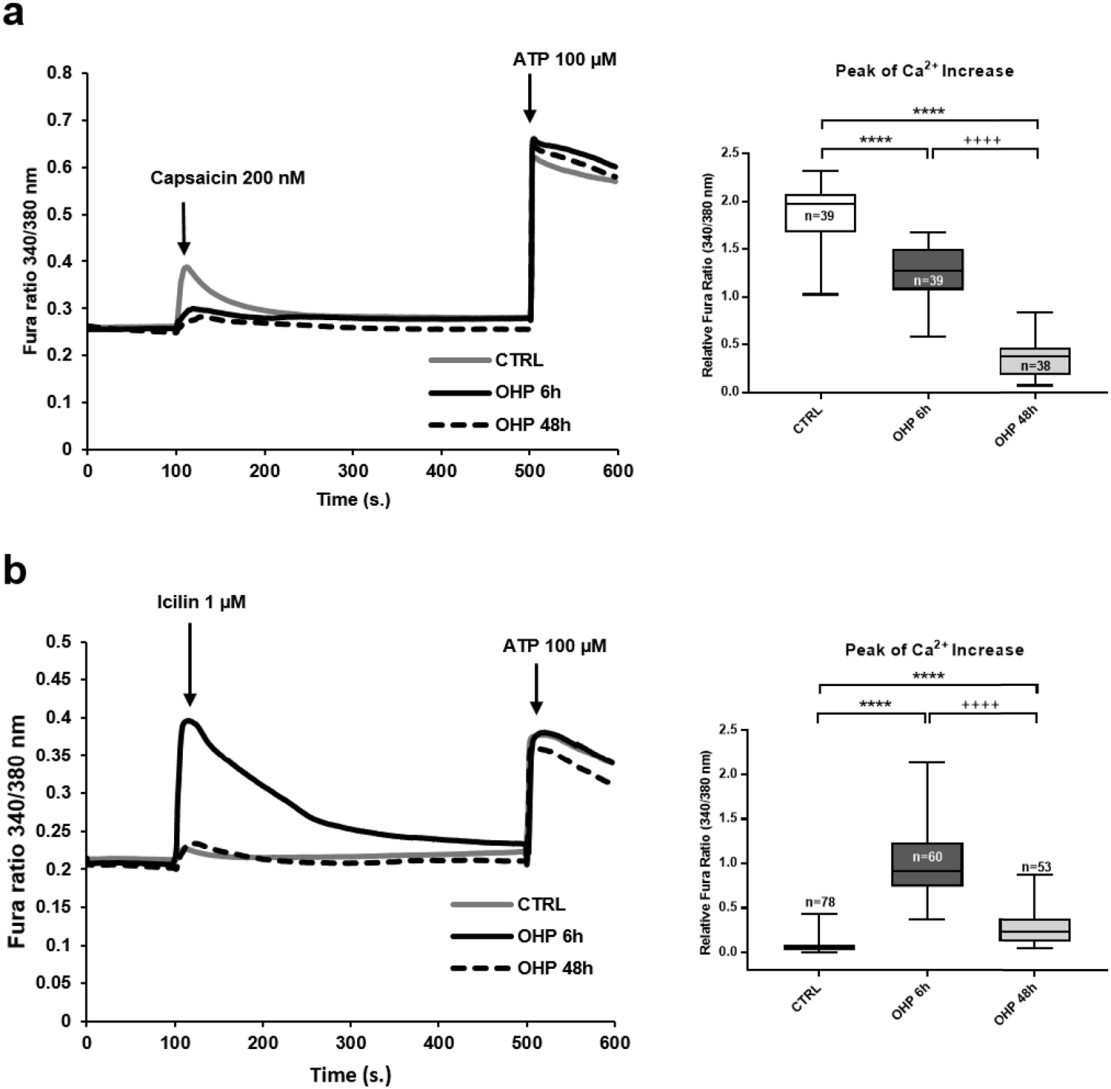
Calcium responses to capsaicin (**a**) and icilin (**b**). DRG neurons treated with OHP (0.1 μg/mL) for 6 or 48 hours or untreated (CTRL) were loaded with Fura2-AM and placed in an extracellular solution containing 2 mM calcium. Traces are the average of the cells indicated in the corresponding bar graphs, obtained from 3 independent experiments. Box and whisker plots show median and IQR of peak of calcium changes. Kruskal-Wallis H test followed by Dunn’s post-hoc. ****P < 10^−6^, ^++++^P < 10^−6^. Calcium responses to AITC can be found in Suppl. Fig. 3.

We first set out to investigate whether the above treatment conditions might affect TRPV1, TRPM8 and TRPA1 responses, in analogy to what observed by others with more aggressive protocols. DRG neurons exposed to OHP for 6 hours (0.1 µg/mL) challenged with capsaicin (TRPV1 agonist; 200 nM) showed a reduced Ca^2+^-response compared to controls (median of CTRL: 1.98; median of OHP: 1.28; ****P = 1.4·10^−5^). The reduction was more evident for neurons exposed for 48 hours to OHP (0.1 µg/mL; median of CTRL: 1.98; median of OHP: 0.37; ****P < 10^−6^; Fig. 1a).

Conversely, only a minority of the control DRG neurons responded to icilin (TRPA1 and TRPM8 agonist; 1 µM) with very small Ca^2+^-rises (Fig. 1b). DRG neurons pre-exposed for 6 hours to OHP, instead, unmasked a large icilin-induced Ca^2+^-rise (median of CTRL: 0.05; median of OHP: 0.92; ^****^P < 10^−6^). DRG neurons treated for 48 hours with OHP also displayed an icilin-induced Ca^2+^-rise although this was reduced compared to the shorter treatment (median of CTRL: 0.05; median of OHP: 0.23; P = 4·10^−6^). Therefore, although the effect was evident both after 6 and 48 hours, the shortest treatment was chosen for all other experiments.

Responses to ATP were not affected by OHP in either treatment duration (Fig. 1a,b), suggesting that cells were healthy and that our results were not a manifestation of an experimental artefact. Nonetheless, given the possibility that part of the effects elicited by OHP for 48 hours might have been attributed to neuronal suffering, we decided to focus our attention on the shorter treatment.

Given that OHP unmasked a response to icilin, we also investigated the neurons that were responsible for this response by measuring their average soma diameter (Suppl. Fig. 2a for distributions). Briefly, our cell cultures were characterized by the presence of small size neurons (median of soma diameter for control neurons of 13.8 µm, n = 278) and similar values were observable in OHP-treated neurons (13.1 µm, n = 223). The few cells that responded to icilin in control conditions (albeit with very small Ca^2+^-rises) had a median soma diameter of 13.1 µm (n = 55) while the cells that responded to icilin in OHP-treated cultures had a median of 12.5 µm (n = 172). There was no statistically significant difference between the groups (Kruskall Wallis test, P = 0.86, Suppl. Fig. 2b). This would suggest that the observed unmasking of a Ca^2+^-response to icilin might be relevant to the clinical manifestations.

### Icilin responses are mediated by TRPA1 and not by TRPM8

**Figure 2:**
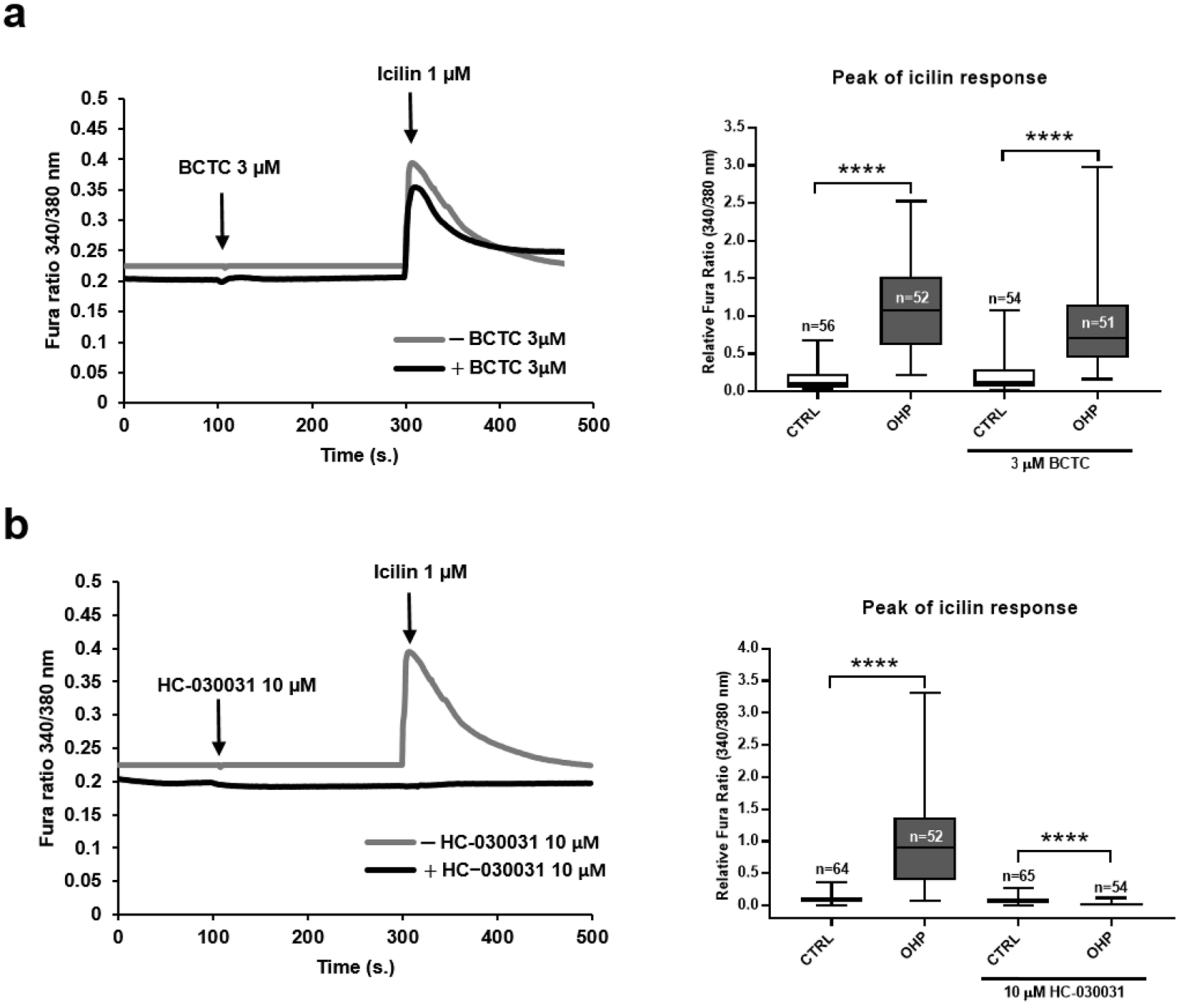
OHP increases icilin responses by a TRPA1-dependent mechanism. DRG neurons were treated with OHP (0.1 μg/mL) for 6 hours, loaded with 5 μM Fura2-AM and placed in an extracellular solution containing 2 mM calcium. Cells were pre-treated with BCTC (TRPM8 antagonist) (**a**) or HC-030031 (TRPA1 antagonist) (**b**) before being challenged with icilin. Traces are the average of the cells indicated in the corresponding bar graphs, obtained from 6 independent experiments. Box and whisker plots show median and IQR of peak calcium responses in the presence or in the absence of BCTC or HC-030031. Mann-Whitney U test; ****P < 10^−6^.

Icilin has been reported to be an agonist of both TRPM8 and TRPA1 receptors^19,20^. To discriminate between the two, we used the specific inhibitors of TRPM8 (BCTC) or TRPA1 (HC-030031). As it can be observed in Fig. 2a, icilin induced a response in OHP-treated DRG neurons both in the presence (3 µM; median: 0.71) or absence of BCTC (median: 1.01) suggesting that the TRPM8 was not involved. On the contrary, the response to icilin was completely abolished in the presence of HC-030031 (10 µM; median: 0.02) in OHP-treated neurons. In accord, in our conditions, preliminary experiments showed that menthol elicited very small responses (data not shown), suggesting that TRPM8 does not contribute to the icilin-response. This might be due to a number of factors, including the temperature at which we perform experiments.

Allyl isothiocyanate (AITC) is another well-known agonist of TRPA1 and we therefore evaluated whether the potentiation of the response by OHP treatment could be unmasked also by activation of the channel by this compound. As shown in Suppl. Fig. 3, in control cells AITC induced only a modest response (median: 0.09), while in OHP-treated neurons the response was significantly potentiated (median: 0.39). The potentiated response was sensitive to HC-030031 (10 µM; median: 0.06).

**Figure 3:**
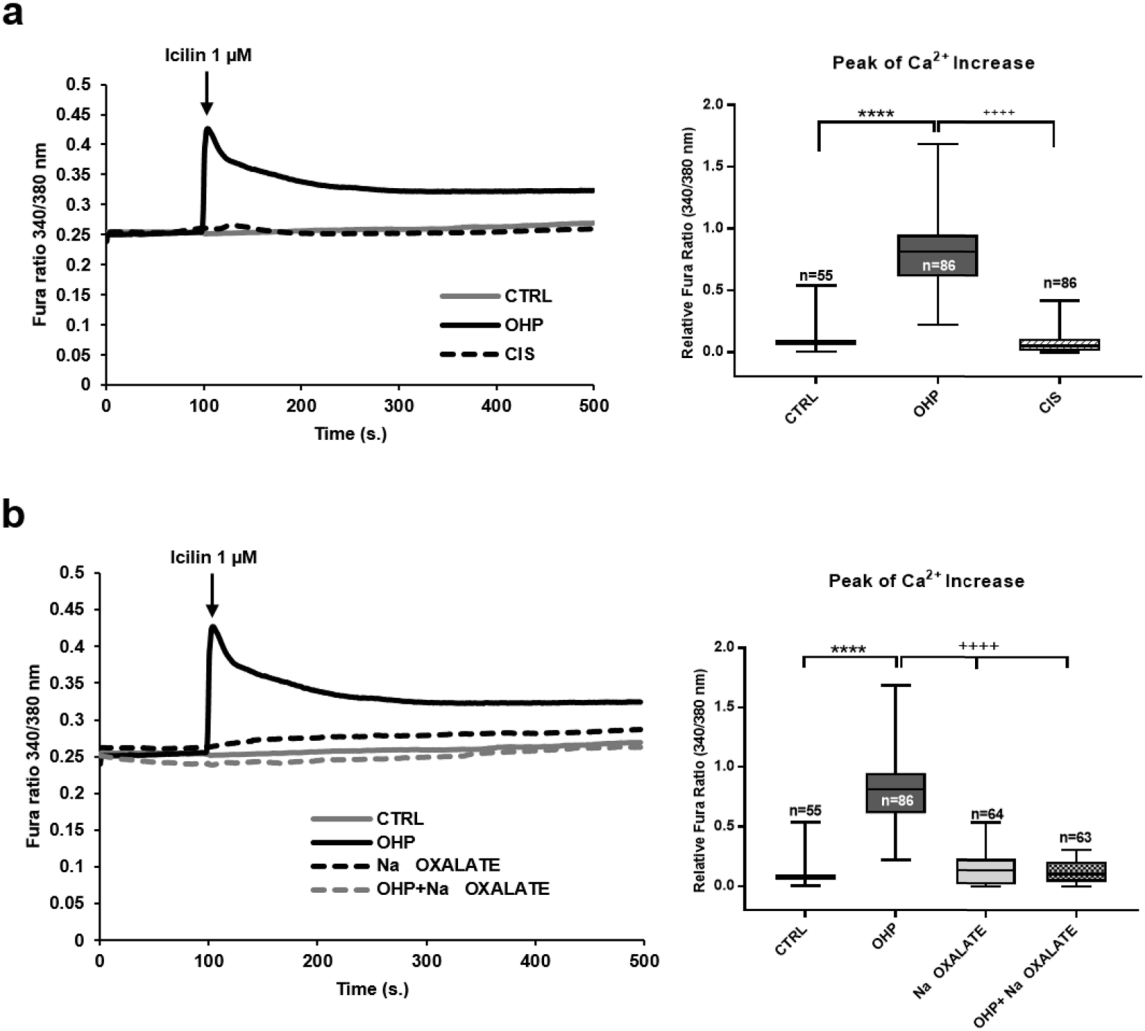
Icilin responses induced by cisplatin (**a**) or sodium oxalate (**b**). DRG neurons were treated with OHP (0.1 μg/mL), cisplatin (CIS; 0.1 μg/mL), or sodium oxalate (0.3 μg/mL) alone or in combination with OHP (0.1 μg/mL) for 6 hours. Traces are the average of the cells indicated in the corresponding bar graphs, obtained from 4 independent experiments. Box and whisker plots show median and IQR of peak of calcium changes after the addition of 1 µM icilin. Kruskal-Wallis H test followed by Dunn’s post-hoc. ****P < 10^−6^ and ^++++^P < 10^−6^.

These data would therefore suggest that, in our model, OHP modulates the response to TRPA1.

### Sodium Oxalate or cisplatin do not mimic the effect of OHP

We next tested whether treatment for 6 hours with the close analogue cisplatin or sodium oxalate were able to mimic the sensitization of TRPA1. As it can be observed (Fig. 3a), cisplatin (0.1 µg/mL; median: 0.05), unlike OHP (median: 0.81), was unable to potentiate the response to icilin in DRG neurons. Similarly, sodium oxalate, at the same molar concentration (0.3 µg/mL), was also unable to unmask a TRPA1-dependent Ca^2+^-response (median: 0.14) (Fig. 3b). We also evaluated whether sodium oxalate was able to modify the response to OHP and, to our surprise, co-treatment with equimolar concentrations of OHP and sodium oxalate led to the abolishment of the icilin response (median OHP: 0.82, median co-treatment: 0.10). Oxalate can chelate Ca^2+^-ions, although it is unlikely that the low concentrations used (0.3 µg/mL; 2.2 µM) are able to mask Ca^2+^-responses, also in light of the mM concentrations of Ca^2+^ in the extracellular medium. To verify that indeed oxalate does not affect Ca^2+^-responses, we evaluated ATP-induced Ca^2+^-mobilization in the same cells. As observed in the Suppl. Fig. S4, the ATP induced Ca^2+^-responses were not different in control, OHP-treated, and oxalate-treated cells (medians: 0.77; 0.91; 0.65, respectively).

The antagonistic effect of oxalate was unexpected and we therefore evaluated whether this also occurred for the cytotoxic effect of OHP. To this end, we treated 6 different colorectal cancer cell lines with OHP (3 µg/mL) in the presence or absence of equimolar concentrations of oxalate (9 µg/mL) for 24, 48 or 72 hours. The concentration of OHP was chosen based on the IC50 obtained in preliminary experiments for the 48 h treatment. OHP was cytotoxic to the same degree in the presence or absence of oxalate (Suppl. Fig. S5), suggesting that this antagonism is specific for TRPA1 calcium response in DRG neurons.

### OHP does not modify mRNA levels for TRPA1 or TRPV1

After the 6 hour treatment with OHP, DRG cells are maintained for a further 42 hours in culture before experiments. Therefore, it is possible that gene expression changes may be responsible for the effects observed. Treatment with OHP did not induce any change in mRNA levels for TRPA1 (1.0 ± 0.1 of control; n = 6), TRPV1 (1.1 ± 0.1 of control; n = 6) or TRPM8.

We also recently performed a microarray analysis with a validation set to investigate gene expression changes upon OHP treatment *in* vivo in DRG cells^14^. When interrogating the microarray results for these two genes, we confirmed that no significant changes occurred for either TRPA1 (|log2FC| 0.142) or TRPV1 (|log2FC| 0.016) *in vivo*. Similarly, RT-PCR data of the confirmation dataset performed on mRNA from DRG cells from a different set of animals confirmed that no significant changes occurred in these animals for these two channels (TRPA1 1.1 ± 0.2, TRPV1 1.0 ± 0.2; both normalized to control; n = 8).

### pH changes occur upon OHP treatment *in vitro* and *in vivo*

If changes in channel expression cannot account for the change in icilin-response observed, *i.e*. the same number of channels are present in control and OHP-primed neurons, other explanations might account for the results observed. It is well known that TRP channels are multimodal receptors, able to detect a number of chemical and physical agents. In this respect, it has been reported that intracellular pH changes are able to modulate in opposite directions the effect of TRPV1 and TRPA1^21^, which would be compatible with our Ca^2+^-signalling results (Fig. 1).

**Figure 4:**
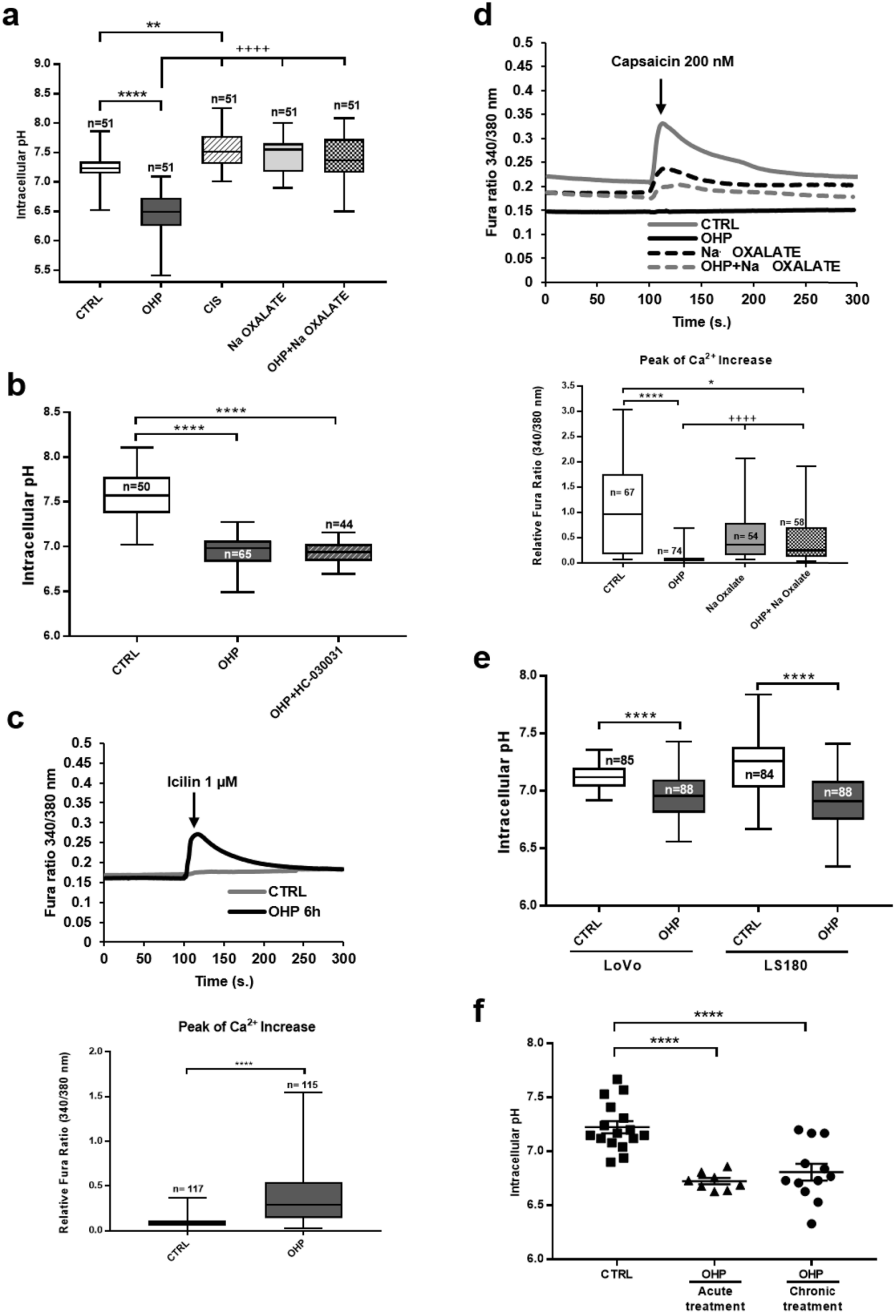
Effects of OHP, cisplatin or sodium oxalate on intracellular pH. (**a**) pH evaluation in cultured DRG neurons treated with OHP (0.1 μg/mL), cisplatin (CIS; 0.1 μg/mL) or sodium oxalate (0.3 μg/mL) alone or in combination with OHP (0.1 μg/mL) for 6 hours. Box and whisker plots show median and IQR of pH_i_ values. For each treatment the number of cell is indicated in the corresponding bar; data are from 7 independent experiments. Kruskal-Wallis test followed by Dunn’s post-hoc. ****P < 10^−6^, **P = 0.0078; ^+++^P = P < 10^−6^ OHP vs. CIS, sodium oxalate and OHP + sodium oxalate; (**b**) pH evaluation of cultured DRG neurons treated with OHP (0.1 μg/mL) in the presence or absence of HC.030031 (10 µM) for 6 hours. Box and whisker plots show median and IQR of pH_i_ values. For each treatment the number of cell is indicated in the corresponding bar; data are from 2 independent experiments. Kruskal-Wallis test followed by Dunn’s post-hoc. ****P < 10^−6^; (**c,d**) Ca^2+^-responses to icilin (1 µM; c) or capsacin (200 nM, d) in cells treated for 6 hours with the indicated compounds (OHP 0.1 µg/ml and oxalate 0.3 µg/ml). Box and whisker plots show median and IQR of peak of calcium changes after the addition of compounds. Kruskal-Wallis test followed by Dunn’s post-hoc. ****P < 10^−6^, *P = 0.031, ^++++^P<10^−6^; (**e**) pH evaluation in the indicated colorectal cancer cells treated with OHP (0.1 μg/mL) for 6 hours and determined at 48 hours. Box and whisker plots show median and IQR of pH_i_ values. For each treatment the number of cell is indicated in the corresponding bar; data are from 2 independent experiments. Kruskal-Wallis test followed by Dunn’s post-hoc. ****P < 10^−6^; (**f**) freshly excised DRG cells from control or acutely or chronically OHP-treated BALB/c mice were loaded with 5 μM BCECF and placed in an extracellular physiological solution. Ratiometric measurements of pH_i_ and calibration curves were obtained by flow cytometry. Graphs show the mean ± S.E.M from n = 16 controls, n = 12 chronic treatment, or n = 8 acutely treated animals. One-way analysis of variance (F = 19.38) followed by Tukey’s post-hoc. ****P < 10^−6^.

To investigate this aspect, we made use of BCECF, a specific ratiometric fluorescent pH probe^22,23^. As it can be observed (Fig. 4a), a significant median drop from 7.23 to 6.49 occurred in cells exposed to OHP (^****^P < 10^−6^). Paralleling the results obtained in calcium signalling (Fig. 3), such changes did not occur upon exposure for the same length of time with either oxalate (7.55) or cisplatin (7.51). In line with what observed for calcium signalling, while devoid of any effect *per se*, oxalate was nonetheless able to revert the effect of OHP (7.36; Fig. 4a).

While our protocol is translational (6-hour treatment followed by 42 hour recovery) it fails to disclose when this pH change takes place, *i.e*. in the first 6 hours or afterwards. To dissect the two, we then performed experiments at 6 hours. As shown in Fig. 4b, 6 hours treatment with OHP was sufficient to reduce pH significantly (medians: control 7.37 and OHP 6.83). The effect on pH at 6 hours was paralleled by the sensitization to icilin (Fig. 4c, median of CTRL: 0.08; median of OHP: 0.28) and the reduction of the capsaicin response (Fig. 4d, median of CTRL: 0.96; median of OHP: 0.06). To confirm that the effects observed at this time-point were linked to those observed at 48 hours, we also co-treated for 6 hours DRG cultures with OHP and oxalate and tested the recovery of TRPV1 responses. As observed in Fig. 4d, oxalate led to a partial recovery of TRPV1 responses, linking the two observations (median of oxalate: 0.36; median of OHP + oxalate: 0.25).

To understand whether pH changes were up-stream or down-stream of TRPA1-sensitization, we treated DRG cultures with OHP in the presence of HC-030031. In this manner, we tested whether acidification is the result of TRPA1 activation. As shown in Fig. 4b, HC-030031 had no effect on OHP-induced acidification (median: 6.84 compared to OHP 6.83), strongly suggesting that cytosolic acidification is an up-stream effect.

To investigate whether these OHP-induced pH changes were specific for DRG neurons or could be observed also in other cells, we tested, with the 48 hours protocol (6 hours treatment and further 42 hours in normal medium), two different colorectal cancer cells, LoVo and LS180. In both cells, OHP-treatment (0.1 µg/ml) led to a significant decrease in pH (medians for LoVo: control 7.12, OHP 6.81; for LS180 control 7.26 OHP 6.91) albeit of smaller entity compared to that observed in DRG neurons (Fig. 4e). This strengthens the notion that in DRG neurons, TRPA1 sensitization is down-stream of acidification, as these cells do not express TRPA1.

All the above data was obtained on DRG neurons or on cancer cell lines in culture, and, to test the relevance of these pH changes we felt necessary to explore the pH of DRG cells from OHP-treated animals. Briefly, BALB/c mice were treated either with OHP (3.5 mg/Kg) twice a week for four weeks^14^ or only once (3.5 mg/Kg). Twenty-four hours after the last treatment, animals were sacrificed, DRG dissected, dissociated and FACS analysis with BCECF was performed as described in the materials and methods section. The average pH of cells (Fig. 4f) from control animals was 7.22 ± 0.05 (n = 16). The average pH of cells from animals treated chronically with OHP was 6.80 ± 0.07 (n = 12) while average pH from animals treated once with OHP was 6.72 ± 0.03 (n = 8). These data therefore correlate with what observed in DRG neurons in culture.

### Restoration of physiological pH suppresses TRPA1 hyper-sensitivity

The above data would suggest that OHP acidifies the pH of DRG cells, both *in vitro* and *in vivo*, and this in turn modifies the activity of pH-sensitive channels, including TRPA1. If this were the case, it would be expected that experimental manipulations that restore pH of DRG neurons would normalize the channel activity.

**Figure 5:**
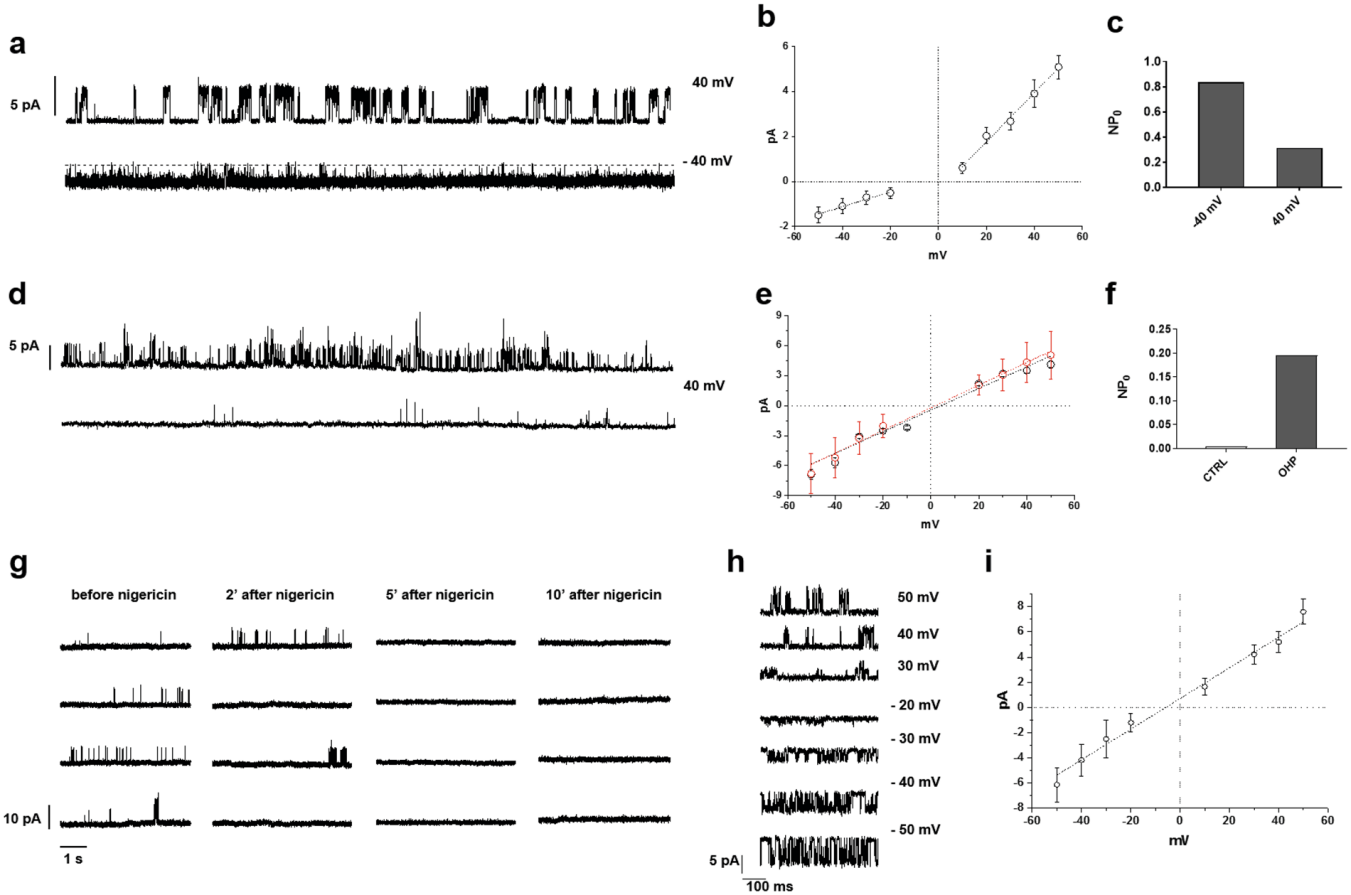
Restoration of physiological pH suppresses TRPA1 hyper-sensitivity to icilin in cell-attached patches. Single channel recordings at two different membrane potentials of a TRPA1 channel obtained in the presence of 2 mM CaCl_2_ in the patch pipette from an OHP treated DRG neuron (**a**). *i-V* plot with linear regressions (**b**) and NP. (**c**) for the same channel shown in (**a**). In this experiment and in all other shown in the figure, the patch pipette contained 1 µM icilin. (**d**) Two example of TRPA1 channel activity recorded in the absence of divalent ions in the patch pipette from an OHP-treated (upper trace) or untreated neuron (lower trace). *i-V* plot with linear regressions (**e**) and NP_o_ (**f**) for the channels shown in (**d**). Red symbols and line refer to treated neuron. (**g**) Single channel traces from an OHP-treated neuron before and during nigericin perfusion, that induces a pH_i_ shift towards a slightly alkaline value. (**h**) Elementary currents recorded for the channels shown in (**g**) at different membrane potentials and the corresponding *i-V* plot with linear regressions (**i**).

To investigate this, we decided to perform electrophysiological patch clamp experiments in the cell attached mode, the recording configuration that less interferes with the intracellular milieu composition. In all experiments, DRG neurons were perfused with a bath solution containing 150 mM KCl and low [Ca^2+^] (see methods), to set the membrane potential near zero and reduce the calcium load. Figure 5a shows two records of 30 s long showing the channel activity in an OHP treated neuron in the presence of icilin (1 µM in the presence of BCTC) and of 2 mM of CaCl_2_ in the pipette solution at V_m_ = 40 mV (upper trace) and −40 mV (lower trace). Single channel currents were outwards at positive voltages and inwards at negative voltages as expected for a non-selective cationic channel. Figure 5b shows the I-V relationship obtained from the same experiment. Single channel conductance (γ) and reversal potential (V_r_) were respectively of 109.3 ± 6.7 pS and of 3.9 ± 1.6 mV for outward currents and of 32.9 ± 3.3 pS and of −5.9 ± 3.4 mV for inward currents. Furthermore, the channel activity (NP_o_, were N is the number of channels in the patch and P_o_ the open probability) was voltage-dependent, with high open probability at negative potentials and a voltage-dependent inactivation at positive potentials (Fig. 5c). These channel properties are in good agreement with previously published data, and confirm that in OHP-treated neurons icilin robustly activates TRPA1 channels^24–29^.

We next addressed the characterization of the channel biophysical properties in the absence of divalent ions in the pipette, with the purpose to attenuate calcium-dependent inactivation^25,28^, a prerequisite for long duration experiments. Figure 5d shows a 30 s recording of the patch currents from an OHP treated neuron in the presence of 1 μM of icilin at Vm = 40 mV (upper trace) in which at least three channels were active. Moreover, the red open circles of Fig. 5e represent the single channel current amplitudes recorded at different membrane potentials. In this condition, the fitted regression line yields a γ and a V_r_ respectively of 107.8 ± 7.6 pS and of 4.2 ± 2.0 mV. The lower trace of Fig. 5d was obtained from an untreated neuron again in the presence of icilin in the pipette. Single channel current amplitudes at different V_m_ are represented as black open circles in Fig. 5e. The values of the elementary conductance and the reversal potential were very similar for the two patches (γ = 113.2 ± 5.0 pS and Vr = 2.0 ± 1.4 mV). Moreover, Fig. 5f compares the channel activity NP_o_ at Vm = 40 mV of the traces in D. We also obtained similar values in two other untreated (NP_o_ = 0.002 and 0.004) and treated neurons (NP_o_ = 0.13 and 0.05). This result suggests that NP_o_ is of one to two orders of magnitude greater in OHP-treated neuron compared to control.

Last, we assessed if the NP_o_ increase observed in treated neurons was dependent on the OHP-induced pH_i_ acidification. For this purpose, we added to the bathing solution 10 μM of the K^+^/H^+^ exchanger nigericin. In our ionic conditions, the insertion in the membrane of the ionophore should activate a net movement of H^+^ outside the cell that will set the pH_i_ value close to the extracellular one. To validate this approach, using BCECF we evaluated the effect of nigericin on intracellular pH in DRG neurons pre-treated for 6 hours with OHP and then left for 42 hours in the same experimental conditions (i.e. high extracellular potassium, low calcium extracellular solution). As shown in Suppl. Fig. S6, the addition of nigericin led to an alkalinisation of intracellular pH, from approx. 6.8 to 7.5. We hypothesized that the change from an acidic to a slightly alkaline pH, should decrease TRPA1 channel activity, if the original hypothesis were true. We observed such effect in all the three neurons tested. In the experiment shown in Fig. 5g and in another one, a complete inactivation of the channel occurred after about 2 min of nigericin perfusion. From the amplitude analysis of single channel recording obtained at different membrane potentials we obtained the I-V relationship (Fig. 5f) for which the elementary conductance and the reversal potential were again very close to the expected values for TRPA1 channel (γ = 121.1 ± 7.0 pS and Vr = −8.4 ± 1.8 mV). A complete channel inactivation after 7 min of nigerin perfusion was also observed in an experiment performed at V_m_ = −40 mV.

## Discussion

In the present contribution we show that therapeutically-relevant OHP concentrations^18^ induce an acidification of intracellular pH both in cultured DRG neurons and in DRG cells from treated animals. This pH change leads to a sensitization of TRPA1 channels which can be reversed by restoring pH to physiological level. Remarkably, acidification occurs even after a single injection of OHP in animals, suggesting its relevance for the occurrence of acute OIPN. This is strengthened by the observation that equi-molar concentrations of cisplatin, *in vitro*, are unable to induce any pH change.

It should be noticed that, while we do not see an effect of cisplatin at low concentrations on pH, higher concentrations (that we have not investigated) may be able to elicit an effect on DRG neurons. Indeed, this has been shown previously on colorectal cancer cells at approx 100-fold the concentrations used in the present study (20–30 µM)^30,31^.

This may be indicative of the fact that all platinum containing drugs share this effect but have different potencies. Indeed, while it could be envisaged that its the oxalate molecule from oxaliplatin that is responsible for the effect observed, we believe this is not the case as: (i) the concentration of released oxalate would be too low; (ii) oxalate surprisingly reverts the effect of oxaliplatin; and (iii) cisplatin, at 100-fold higher concentrations, can elicit similar effects. It is interesting to speculate that the different relative potencies between the cytotoxic and pH effect may be responsible for the different neurotoxic potential of the drugs. In the present contribution, we indeed provide evidence that the effect on pH also occurs in colorectal cancer cells, at lower concentrations compared to the cytotoxic effect, thereby possibly dis-joining the two effects.

It should be noticed that the concentrations *in vitro* in the present manuscript are lower than those of previous reports attempting to elucidate the mechanism of OIPN^16,17^, and therefore they are not in contradiction. Nonetheless, we believe that these lower concentrations more closely resemble those found in plasma of patients and of mice treated with OHP^32^ compared to higher concentrations used in the literature.

In our hands, equimolar concentrations of oxalate were also unable to induce intracellular pH changes. Yet, to our surprise, co-treatment of equimolar concentrations of oxalate were able to abolish OHP-induced pH changes. At present, no explanation can be put forward for this, although it demonstrates that OHP-induced pH acidification occurs via a specific and saturable mechanism. It should be noted that oxalate antagonized the change in pH (and the relative sensitization of the channel), but did not antagonize the cytotoxic effects of OHP in any of the 6 colorectal cell lines tested, suggesting that the site of interaction might represent an exploitable pharmacological target in DRG to reduce OIPN. However, since oxalate is *per se* toxic, it is unlikely that it could be directly used for OIPN prevention. In fact, oxalate is mainly nephrotoxic^33^, and, at high doses, neurotoxic^11,34^.

Our data are partly in contrast with earlier evidence that oxalate and OHP produce similar neurotoxic effects in rodents^34^. It should be nonetheless noticed that whether these two agents act via the same mechanism has never been investigated.

In the present contribution we have focused on TRPA1, a TRP channel that has been shown earlier to be involved in OIPN. For example, pharmacological inhibition of TRPA1 abolishes OHP-induced mechanical and cold-hypersensitivity and this effect is also abolished in TRPA1-null mice^12,35,36^. Furthermore, hypersensibility has also been shown upon pre-treatment *in vitro* in DRG neurons, in agreement with our data^12,36^. Remodelling of TRPA1 expression has been postulated as one the mechanisms that may underlie this hypersensitivity^13,16^, but our data, obtained both *in vitro* and *in vivo*, failed to detect any mRNA change related to this channel^14^ and this report. It has also been recently pointed out that while channel remodelling may explain chronic OIPN, it is unlikely that protein changes may explain hypersensitivity occurring within hours of exposure^36^.

Our observation that intracellular pH acidification hyper-sensitizes the TRPA1 channel is supported by earlier data that report intracellular pH-dependent activation of this channel^37,38^. Indeed, this was postulated to be the mechanism by which humans detect some of the sensations from carbonated beverages^39^. Yet, it is likely that intracellular pH changes do not affect solely this channel, as other channels may also be affected. Indeed, TRPM8, the other TRP family member that has been postulated to be responsible for acute OIPN, has also been demonstrated to display pH sensitivity, although in this context acidification led to a decreased sensitivity^40^. This observation is supported by the modest icilin-dependent BCTC-sensitive response in OHP-treated DRG neurons we observed. Also, TRPV1 has been shown^37^ to be affected negatively by intracellular pH acidification, which is in agreement with the data obtained in the present manuscript (Fig. 1).

In conclusion, our results suggest the OHP-induced cytosolic acidification as one of the key factors involved in the positive modulation of TRPA1 channels activity and this is related at least to the acute OHP neurotoxicity.

## Electronic supplementary material

Supplementary Information
