# Supplementary material for: Oxaliplatin induces pH acidification in dorsal root ganglia neurons
Source: Sci Rep. 2018 Oct 10;8:15084. doi: 10.1038/s41598-018-33508-6 (PMC6180129; doi:10.1038/s41598-018-33508-6)
Supplement: Supplementary file 1 — Supplementary Information [file 41598_2018_33508_MOESM1_ESM.pdf]

Oxaliplatin induces pH acidification in dorsal root ganglia neurons

Riva B.[1], Dionisi M.[1], Potenzieri A.[1], Chiorazzi A.[2], Cordero-Sanchez C[1], Rigolio R.[2], Carozzi VA.[2,3], Lim D.[1], Cavaletti G.[2], Marmiroli P.[2], Distasi C.[1], Genazzani AA*[1].

[1] Department of Pharmaceutical Sciences, University of Piemonte Orientale, Via Bovio 6, 28100, Novara, Italy.

[2] Experimental Neurology Unit, School of Medicine and Surgery, University of Milano-Bicocca, Via Cadore 48, 20900, Monza, Italy.

[3] Young Against Pain Group, Italy

*Address correspondence to:
Prof. Armando Genazzani
Dept. of Pharmaceutical Sciences, University of Piemonte Orientale;
Via Bovio 6,
28100 Novara Italy
Phone ++39 0321 375827 Fax ++39 0321 375821
Corresponding to: armando.genazzani@uniupo,it

Figure S1

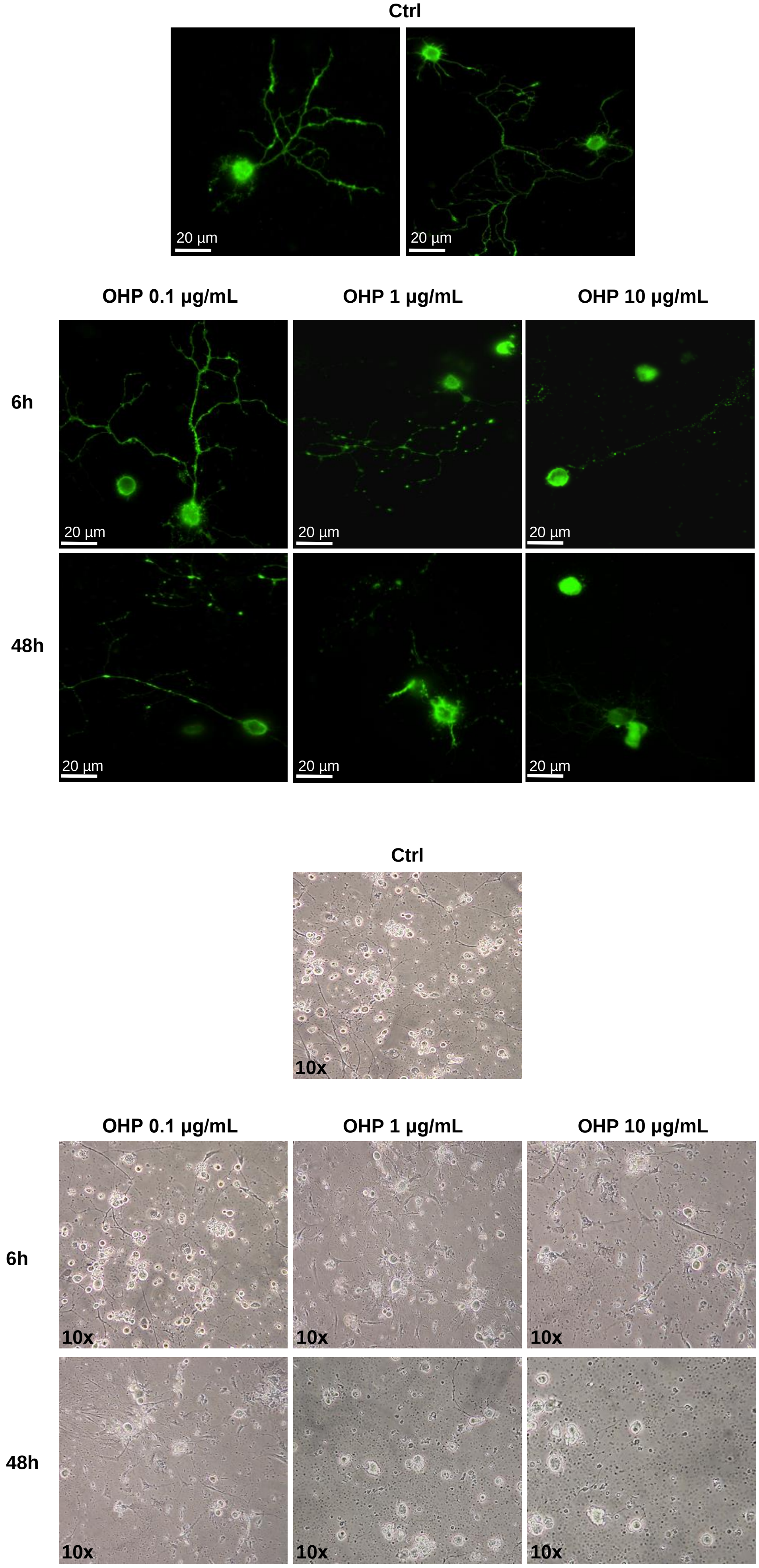
Ctrl
20 µm
20 µm
OHP 0.1 µg/mL
OHP 1 µg/mL
OHP 10 µg/mL
6h
48h
20 µm
Ctrl
10x
OHP 0.1 µg/mL
OHP 1 µg/mL
OHP 10 µg/mL
6h
48h
10x

Figure S2

a

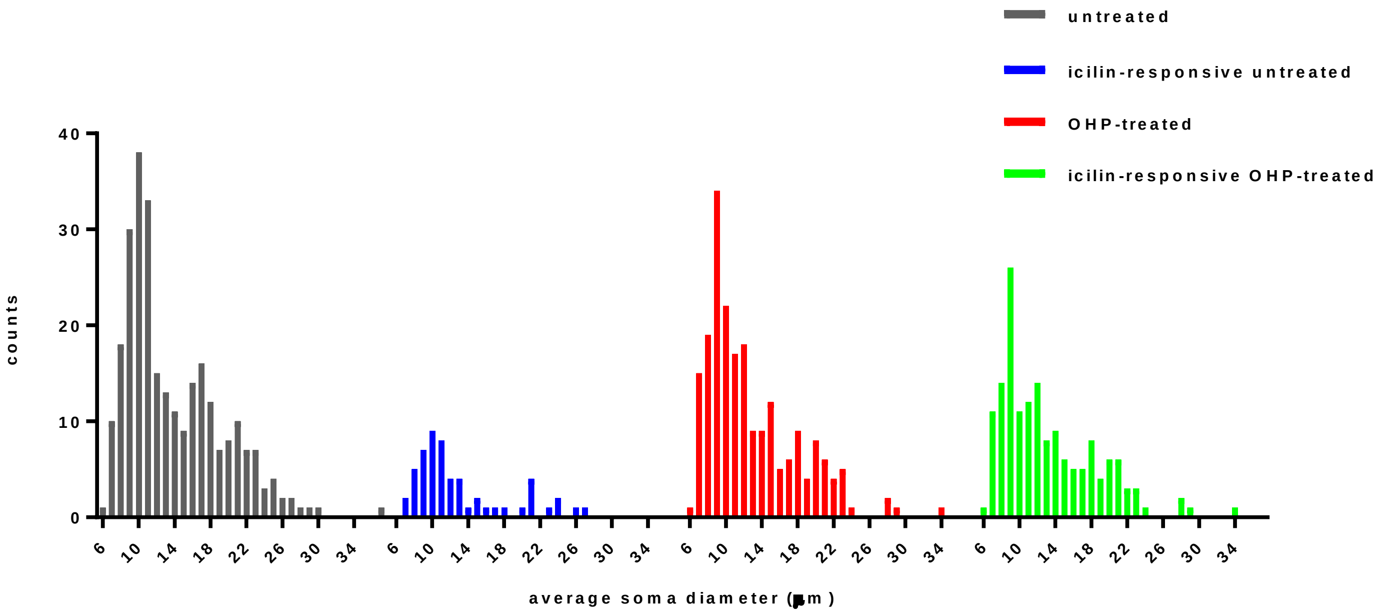
untreated
icilin-responsive untreated
OHP-treated
icilin-responsive OHP-treated
counts
0
10
20
30
40
6 10 14 18 22 26 30 34
6 10 14 18 22 26 30 34
6 10 14 18 22 26 30 34
6 10 14 18 22 26 30 34
average soma diameter (µm)

b

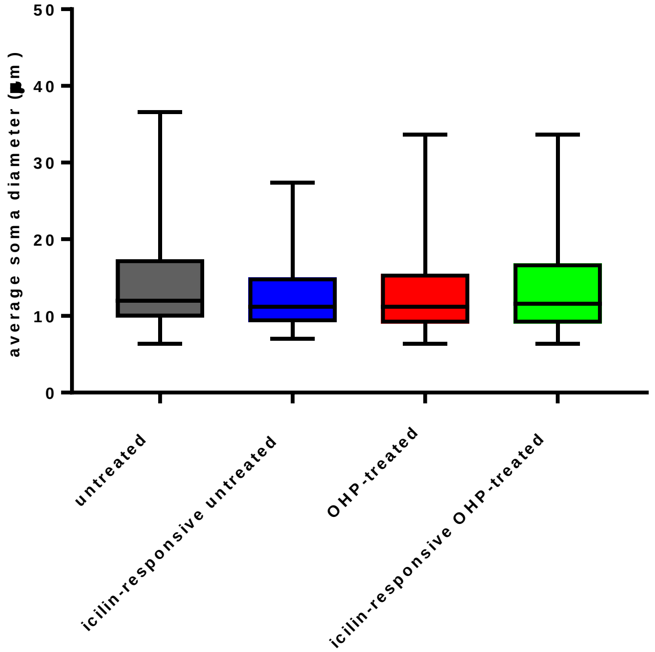
average soma diameter (µm)
0
10
20
30
40
50
untreated
icilin-responsive untreated
OHP-treated
icilin-responsive OHP-treated

Figure S3

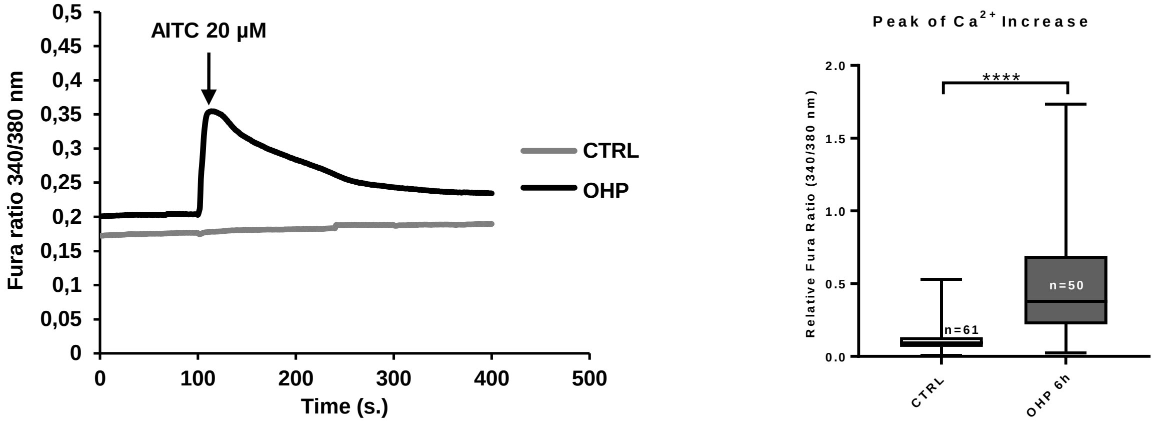
AITC 20 µM
Fura ratio 340/380 nm
0,5
0,45
0,4
0,35
0,3
0,25
0,2
0,15
0,1
0,05
0
0
100
200
300
400
500
Time (s.)
CTRL
OHP
Peak of Ca2+ Increase
Relative Fura Ratio (340/380 nm)
2.0
1.5
1.0
0.5
0.0
****
n=61
n=50
CTRL
OHP 6h

Figure S4

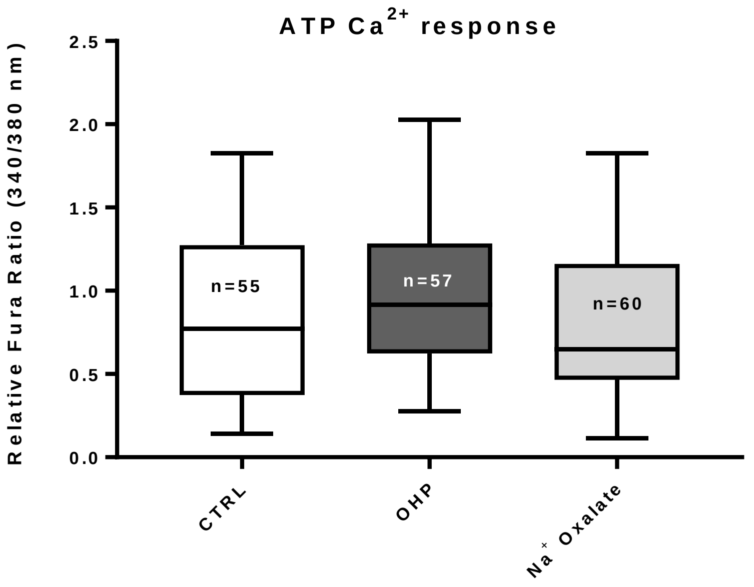

ATP Ca2+ response
Relative Fura Ratio (340/380 nm)
2.5
2.0
1.5
1.0
0.5
0.0
n=55
n=57
n=60
CTRL
OHP
Na+ Oxalate

Figure S5

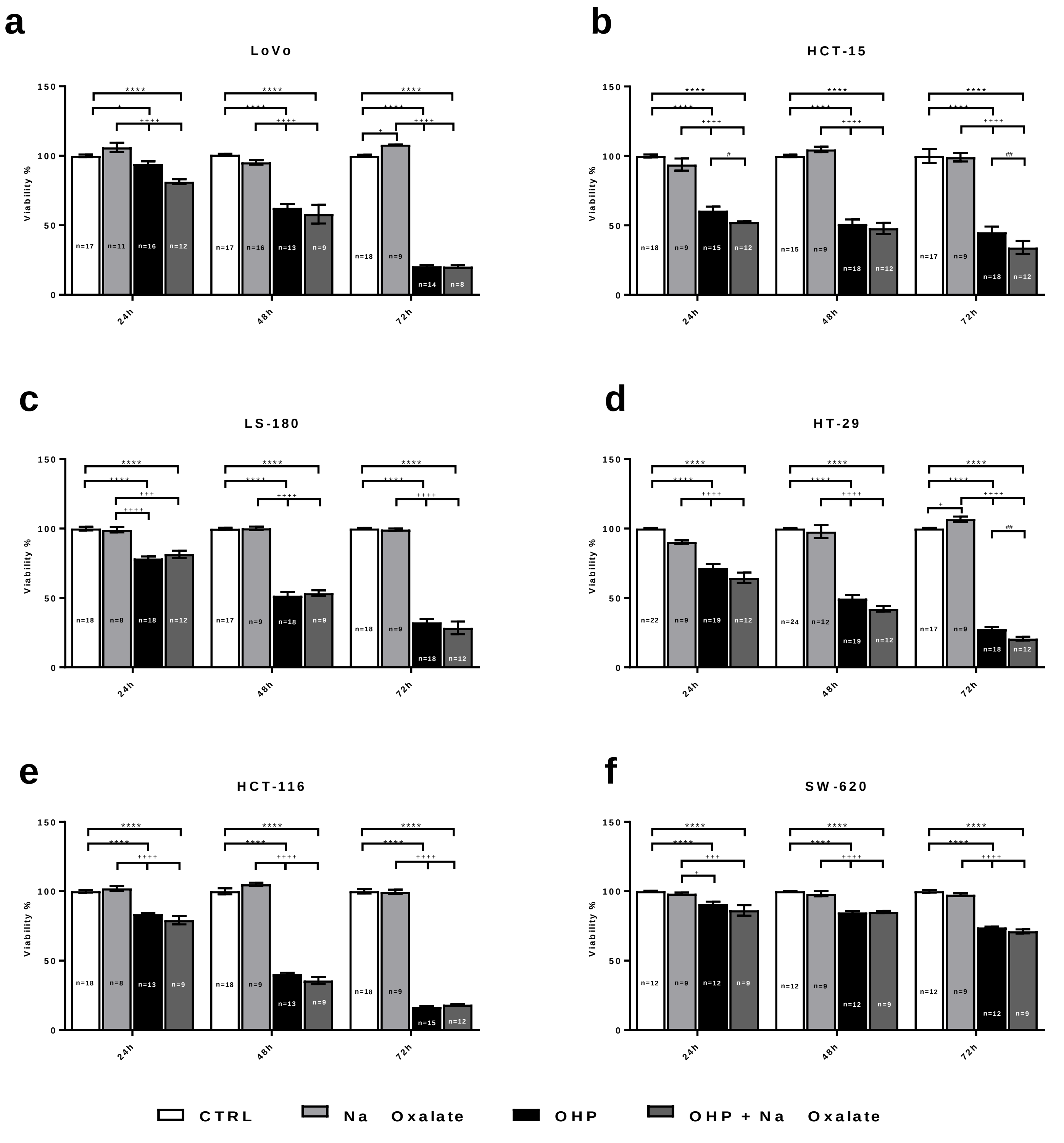

Cell lines	*P* values legend			
**LoVo**	$^{****}P<10^{-6}$	$^{*}P=0.0117$	$^{++++}P<10^{-6}$	$^{++}P=0.0015$
**HCT-15**	$^{****}P<10^{-6}$	$^{++++}P<10^{-6}$	$^{\#\#}P=0.0015$	$^{\#}P=0.040$
**LS-180**	$^{****}P<10^{-6}$	$^{++++}P<10^{-6}$		
**HCT-116**	$^{****}P<10^{-6}$	$^{++++}P<10^{-6}$		
**HT-29**	$^{****}P<10^{-6}$	$^{++++}P<10^{-6}$	$^{+}P=0.021$	$^{\#\#}P=0.0028$
**SW-620**	$^{****}P<10^{-6}$	$^{++++}P<10^{-6}$	$^{+++}P=0.0005$	$^{+}P=0.027$

Figure S6

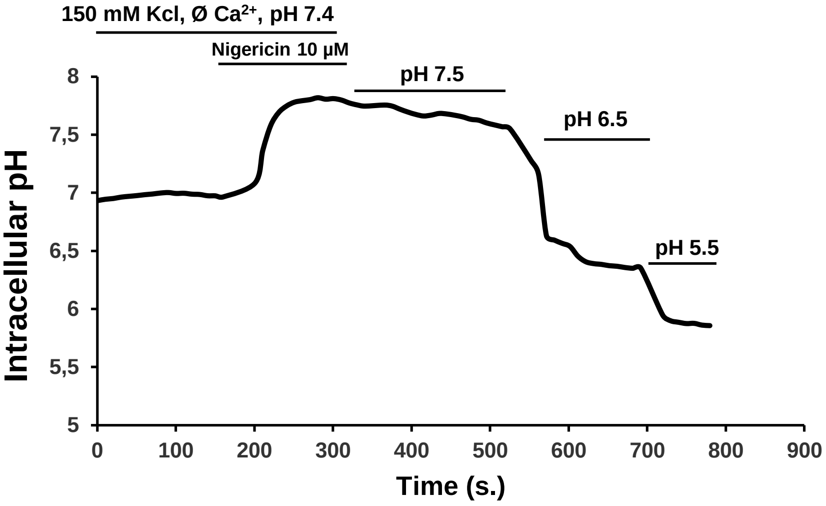

150 mM Kcl, Ø Ca²⁺, pH 7.4
Nigericin 10 µM
pH 7.5
pH 6.5
pH 5.5
Intracellular pH
8
7,5
7
6,5
6
5,5
5
0
100
200
300
400
500
600
700
800
900
Time (s.)

Figure S1. Representative images of DRG neurons cultures.
Immunostainings for GAP-43 in DRG neurons treated at the indicated conditions (top) and representative bright field images (bottom).

Figure S2. Average soma diameter profile of OHP treated and untreated DRG neurons. (**a**). Frequency distributions of average soma diameter for untreated neurons (gray bars), untreated neurons that respond to icilin (blue bars), treated neurons (red bars) and for treated neurons that respond to icilin (green bars). (**b**) Box and whisker plot showing the average soma diameter range and median for the 4 groups showed in A. Kruskal-Wallis H test, P>0.99.

Figure S3. Calcium responses to AITC
DRG neurons treated with OHP (0.1 µg/mL) for 6 hours or untreated (Ctrl) were loaded with Fura 2-AM and placed in an extracellular solution containing 2 mM calcium. Traces are the average of the cells indicated in the corresponding bar graphs, obtained from 5-7 independent coverslips. Box and whisker plots show median and IQR of peak of calcium changes. Mann-Whitney U test; **** P< 10^{-6}.

Figure S4. Calcium responses to ATP in the presence of OHP (0.1 µg/ml) or sodium oxalate (0.3 µg/ml). Data obtained from 3-5 independent experiments. Box and whisker plots show median and IQR of peak of calcium changes. Kruskal-Wallis H test, P= 0.17 ctrl vs OHP; P>0.99 ctrl vs oxalate; P=0.08 OHP vs oxalate.

Figure S5. Cytotoxicity of OHP (3 µg/mL) in the indicated cells lines in the presence or absence of sodium oxalate (9 µg/mL). Histograms show the mean ± S.E.M from three separate experiments. One-way analysis of variance followed by Tukey's post-hoc. At the bottom, the legend for statistical significance is shown.

Figure S6. Effect of nigericin on intracellular pH, determined by BCECF, in high potassium extracellular solutions (see text). OHP-treated DRG neurons were challenged with nigericin and pH standard solutions were then added to the neurons for calibration.
